# Anti-HIV-1 integrase potency of methylgallate from *Alchornea cordifolia* using *in vitro* and *in silico* approaches

**DOI:** 10.1038/s41598-019-41403-x

**Published:** 2019-03-18

**Authors:** Xavier Siwe-Noundou, Thommas M. Musyoka, Vuyani Moses, Derek T. Ndinteh, Dumisani Mnkandhla, Heinrich Hoppe, Özlem Tastan Bishop, Rui W. M. Krause

**Affiliations:** 1 Department of Chemistry, Rhodes University, Grahamstown, 6140 South Africa; 2 Research Unit in Bioinformatics (RUBi), Department of Biochemistry and Microbiology, Rhodes University, Grahamstown, 6140 South Africa; 3 Department of Applied Chemistry, University of Johannesburg, Doornfontein, Johannesburg 2028 South Africa; 4 Department of Biochemistry and Microbiology, Rhodes University, Grahamstown, 6140 South Africa

## Abstract

According to the 2018 report of the United Nations Programme on HIV/AIDS (UNAIDS), acquired immune deficiency syndrome (AIDS), a disease caused by the human immunodeficiency virus (HIV), remains a significant public health problem. The non-existence of a cure or effective vaccine for the disease and the associated emergence of resistant viral strains imply an urgent need for the discovery of novel anti-HIV drug candidates. The current study aimed to identify potential anti-retroviral compounds from *Alchornea cordifolia*. Bioactive compounds were identified using several chromatographic and spectroscopic techniques and subsequently evaluated for cytotoxicity and anti-HIV properties. Molecular modelling studies against HIV-1 integrase (HIV-1 IN) were performed to decipher the mode of action of methylgallate, the most potent compound (IC_50_ = 3.7 nM) and its analogues from ZINC database. Cytotoxicity assays showed that neither the isolated compounds nor the crude methanolic extract displayed cytotoxicity effects on the HeLa cell line. A strong correlation between the *in vitro* and *in silico* results was observed and important HIV-1 IN residues interacting with the different compounds were identified. These current results indicate that methylgallate is the main anti-HIV-1 compound in *A. cordifolia* stem bark, and could be a potential platform for the development of new HIV-1 IN inhibitors.

## Introduction

Acquired immune deficiency syndrome (AIDS), a disease caused by the human immunodeficiency virus (HIV), remains a significant public health problem with more than 76.1 million people infected since its discovery in 1981^1,2^. According to the 2018 report of the United Nations Programme on HIV/AIDS (UNAIDS), about 37 million people are currently living with HIV/AIDS globally with an estimated 5,000 new infections occurring each day, the majority being in sub-Saharan Africa^3^. Upon infection, the virus targets host dendritic and CD4^+^ T cells, subsequently weakening their cell-mediated immunity^4^. Ultimately, this primes the host body systems for attack by a myriad of opportunistic infections and cancers^5^.

Presently, there is no cure or effective vaccine for the disease, mainly due to latency and quiescence inherent in the nature of the virus^6^. However, a customized cocktail of diverse classes of drugs commonly referred to as the highly active antiretroviral therapy (HAART) is available for the management of the disease^7,8^. The different HAART components suppress the replication of the virus in host cells by targeting different stages of its replication cycle: (i) viral entry (co-receptor antagonists and fusion inhibitors); (ii) viral cDNA synthesis [reverse transcriptase (RT) inhibitors]; (iii) viral cDNA-host DNA integration [integrase (IN) inhibitors]; (iv) virion release and maturation [protease (PR) inhibitors]^9^. Despite the considerable success realized hitherto through HAART, the continued emergence of cross-resistant viral strains and associated adverse effects of most of the drugs on patients remain significant challenges to a sustained combat against the disease^10–14^. Thus, there is an urgent need for the discovery of novel anti-HIV drug candidates with improved potency, pharmacokinetic profiles and minimal side effects. Out of the close to 40 HAART drugs approved by the US Food and Drug Administration (FDA), only three target HIV-1 IN *viz*. Raltegravir, Elvitegravir and Dolutegravir^8^. This deficit was initially linked to the inadequate knowledge on the enzyme’s catalytic mechanism and lack of structural information due to its poor solubility and dynamic nature^15^. Mammalian cells lack functional or structural IN homologues, making it a desirable drug target for antiretroviral drug development^16,17^.

The HIV-1 IN enzyme is a 288-amino acid protein with three domains: N-terminal domain^18^ (NTD; residues 1–49); the catalytic core domain (CCD; residues 50–212)^19^ and the C-terminal domain (CTD; residues 213–288)^20^. It plays an essential role in the viral replication cycle by covalently integrating pro-viral cDNA into the host cell’s chromosomal DNA via a two-step S_N_2 nucleophilic reaction: a 3′-end viral cDNA processing and the strand transfer reaction. In the cytoplasm, the enzyme removes two or three nucleotides from the 3′ end of linear pro-viral cDNA at the U3 and U5 ends, exposing the active 3′-OH groups forming a pre-integration complex (PIC)^21^. The PIC is then imported into the nucleus, and IN catalyzes the attack of the exposed 3′-OH groups by phosphate groups of host DNA^18–20,22^. The two independent viral cDNA integration reactions occur in the CCD and are mediated by a DDE motif highly conserved in all retroviruses. Mutagenesis experiments involving the Asp64-Asp116-Glu152 catalytic triad residues inactivated the enzyme as well as virus replication^23^. The Asp residues co-ordinate a divalent cation (Mg^2+^ or Mn^2+^) which is an essential cofactor that facilitates a nucleophilic attack on the 3′-OH groups and stabilization of transition states^19,24^.

**Figure 1:**
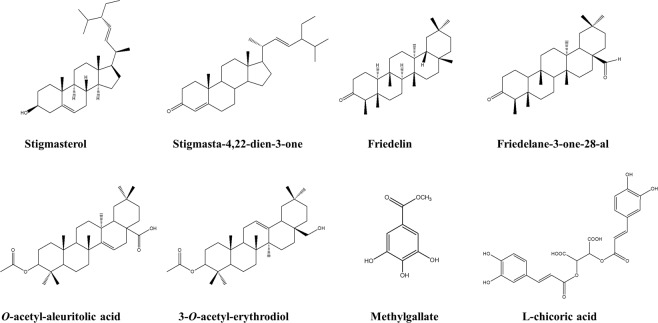
Chemical structures of isolated compounds (1–7) from *A. cordifolia* and _L_-chicoric acid.

As part of our effort to identify potential anti-retroviral hits, current work evaluates the HIV-1 IN inhibitory properties and cytotoxicity properties of compounds from *Alchornea cordifolia* stem bark using the recombinant form of the enzyme and human HeLa cells respectively. *A. cordifolia* is a tropical medicinal plant distributed throughout central, western, eastern and southern Africa. Its crude extract has been reported to exhibit strong anti-HIV^25^, anti-bacterial^26^ and anti-inflammatory^27^ activities. Out of the seven compounds (Fig. 1) isolated from a crude methanolic extract (MeOH) of *A. cordifolia* stem bark, methylgallate was found to significantly inhibit HIV-1 IN *in vitro* with an IC_50_ value lower than that of _L_-chicoric acid (a known HIV-1 IN inhibitor). Cytotoxicity assays showed that neither the isolated compounds nor the crude methanolic extract displayed cytotoxicity effects on the HeLa cell line. To decipher the mode of action of methylgallate and its analogues from the Zinc Is Not Commercial (ZINC)^28^ database, molecular modelling studies were performed. A strong correlation between the *in vitro* and *in silico* results was observed, and critical HIV-1 IN residues interacting with the different compounds were identified. Molecular dynamic studies to determine the system stability and flexibility revealed that the different IN-ligand complexes investigated in this study were energetically stable. The results presented here indicate that methylgallate is the main anti-HIV-1 compound in *A. cordifolia* crude extract and could be a potential platform for the development of newer HIV-1 IN inhibitors.

## Methods

### Plant identification and collection

Fresh stem bark of *A. cordifolia* was collected from uncultivated farmland on the Elounden Mountain in the central region of Cameroon in January 2010. Plant specimens were identified by Victor Nana, a botanist at the National Herbarium of Cameroon in Yaoundé. A voucher specimen (Nos. 40512/HNC) deposited in the same herbarium.

### Extraction and identification of compounds

Plant material was dried at ambient temperature and finely ground using a blender. Extraction with methanol was performed by maceration for 72 h at room temperature. Four liters of methanol was used to extract one kilogram of the dry stem bark resulting to 140 g of crude extract. The crude extract was subjected to vacuum column chromatography followed by several open column chromatography steps to yield the pure compounds according to an already established protocol^26^.

### Cell viability assay

The cytotoxicity of the crude extract and isolated pure compounds was evaluated by incubating 20 μM of pure compounds and 25 μg/mL of extracts in 96-well plates containing HeLa (human cervical adenocarcinoma) cells. The cells were maintained in a culture medium made of Dulbecco’s Modified Eagle’s Medium (DMEM) with 5 mM L-glutamine (Lonza), supplemented with 10% foetal bovine serum (FBS) and antibiotics (penicillin/streptomycin/fungizone - PSF) for 24 h. Cell viability was determined by adding resazurin to a final concentration of 0.09 mM to the cells, incubating for 4 hours and quantifying resorufin fluorescence (Excitation_560_/Emission_590_) in a multiwell plate reader.

### Anti-HIV IN assay

The HIV-1 IN strand transfer inhibition assay was adapted from previously described methods^29^. Briefly, 20 nM double-stranded biotinylated donor DNA (5′-5BiotinTEG/ACCCTTTTAGTCAGTGTGGAAAATCTCTAGCA-3′ annealed to 5′-ACTGCTAGAGATTTTCCACACTGACTAAAAG-3′) was immobilized in wells of streptavidin-coated 96-well microtiter plates (R&D Systems, USA) by incubation at room temperature for 40 minutes in phosphate-buffered saline (PBS) containing 0.05% (v/v) Tween 20 and 0.01% (w/v) bovine serum albumin (BSA). After a stringent wash step, 5 µg/ml purified recombinant HIV-1 subtype C IN in integrase buffer 1 (50 mm NaCl, 25 mM Hepes, 25 mM MnCl_2_, 5 mM β-mercaptoethanol, 50 µg/ml BSA, pH 7.5) was added to individual wells. Recombinant HIV-1 subtype C IN was assembled onto the pre-processed donor DNA through incubation for 45 minutes at room temperature, followed by washing. Test compounds and _L_-chicoric acid were added to individual wells as 10-fold serial dilutions starting at 100 µM. Strand transfer was initiated through the addition of 10 nM (final concentration) double-stranded FITC-labelled target DNA (5′-TGACCAAGGGCTAATTCACT/36-FAM/-3′ annealed to 5′-AGTGAATTAGCCCTTGGTCA-/36-FAM/-3′) in integrase buffer 2 (same as buffer 1, except 25 mm MnCl_2_ was replaced with 2.5 mm MgCl_2_). After an incubation period of 60 minutes at 37 °C, the plates were washed using PBS containing 0.05% Tween 20 and 0.01% BSA, followed by the addition of peroxidase-conjugated sheep anti-FITC antibody (Thermo Scientific, USA) diluted 1:1000 in the same PBS buffer. Finally, the plates were washed and peroxidase substrate (SureBlue Reserve™, SeraCare, USA) was added to allow for detection at 620 nm using a Synergy MX (BioTek^®^) plate reader. Absorbance values were converted to % enzyme activity relative to the readings obtained from control wells (enzyme without inhibitor). For IC_50_ determination, dose-response plots of log[compound] vs. % enzyme activity were prepared and IC_50_ values calculated by non-linear regression analysis. Data was fitted to the curve using the least squares method and with a maximum number (1000) of iterations. These calculations were performed, and figures prepared, in GraphPad Prism (version 5.02).

### Homology modelling, molecular docking and dynamic studies

Even with the central function of HIV-1 IN, a complete structure of the functional intasome is unavailable. However, structures of the separate domains have been resolved. From the protein database (PDB), the structure of the catalytic core domain (CCD) of HIV-IN (PDB ID: 1QS4)^30^ co-crystallised with an inhibitor, 1-(5-chloroindol-3-yl)-3-hydroxy-3-(2H-tetrazol-5-yl)-propenone (5-CITEP) and Mg^2+^ ions as a cofactor was retrieved. The structure has four (141–144) missing residues within the 140 s loop which are crucial for its catalytic function. Therefore, 100 model structures were modelled using MODELLER version 9.18^31^ with 1QS4 as the template. Models had the same amino acid sequence as the recombinant protein used for *in vitro* HIV-IN assays. Models were then ranked according to their normalized discrete optimized protein energy (z-DOPE)^32^. The top three models were further validated using ProSA^33^, Verify3D^34^, QMEAN^35^ and PROCHECK^36^ and the best quality model selected for docking studies. AutoDock4.2^37^ was used to determine the interaction and binding affinity of the different compounds. The receptor and compound structure files were prepared in AutoDock4.2 for docking using a previously established protocol^38^. The Gasteiger-Hückel method was used to assign charges on the different ligand and receptor atoms. The Mg^2+^ charge was set at 1.7 as previously determined by the RESP method^38^ and was included in the resulting AutoDock protein file. A cubic grid box with centre at −18.6, 30.1, 66.6 and size of 60 points along the x, y and z directions with point spacing of 0.375 Å was set on the protein. A 100 poses docking simulation run for each ligand was performed using the Lamarckian Genetic Algorithm (LGA). The best docked ligand pose was selected based on clustering and with the lowest docking energy score for molecular dynamics (MD) simulation studies. A 60% similarity search was then used to retrieve analogues of the best compound from the ZINC database. Docking studies on these compounds were performed according to the same protocol.

Seven all-atom Molecular Dynamics (MD) simulations were prepared for the apo IN protein and for ligand-protein complexes showing *in silico* potency with a greater significance than _L_-chicoric acid. All simulations were performed using the Chemistry at HARvard Macromolecular Mechanics (CHARMM 42) software package^39^. For each system, 50 ns MD simulations were created in a 90 Å^3^ cubic box with periodic boundary conditions. Energy minimization was then performed in two stages. The first stage involved the use of a 100 steps of steepest descent minimization *in vacuo*. The second step was 1,000 steps of Adopted Basis Newton-Raphson (ABNR) with a tolerance of 0.001. Each MD system was solvated with TIP3P^40^ water molecules and counter ions were used for neutralization. The solvated system was then minimized using 200 steps of steepest descent followed by 1000 steps of ABNR. Equilibration of the protein was performed by heating the system from 110 K to 310 K. Heating was performed for 100,000 steps using a 1 fs (0.001) time-step. The actual MD simulations were performed at constant pressure and temperature (CPT) conditions. Each 50 ns simulation was achieved by performing 25^7^ steps using a 0.002 time-step. Parameters for describing the protein were obtained from the CHARMM 36 force field^41^. Due to the presence of a Mg^2+^ ion in the active HIV IN, previously determined force field parameters were required to ensure the correct coordination geometry of the active site^38^. These force field parameters were used to account for the bonds and dihedral angles crucial for the residues coordinating the Mg^2+^ active site. The ligands used in the MD simulations were parameterised using the ParamChem webserver^42^ using the CHARMM General Force Field (CGENFF)^43^.

## Results and Discussion

### The crude methanolic extract of *A. cordifolia* stem bark and some of its constituents lack cytotoxicity against HeLa cells

**Figure 2:**
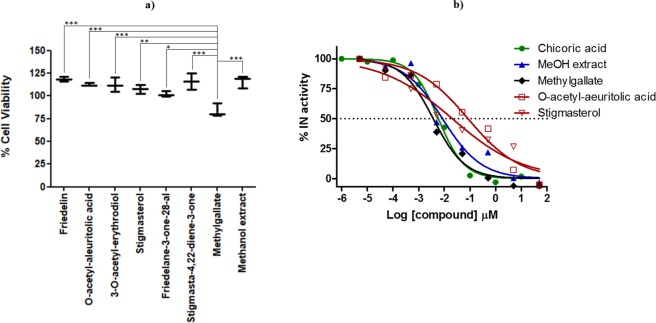
(**a**) The effects of isolated compounds and crude extract of *A. cordifolia* on HeLa cell viability. A vertical box and whisker plot shows the range of cell viability data values (n = 3) and the mean cell viability is marked by the horizontal bar. Statistical analyses showed that the data was normally distributed so a one-way ANOVA and a Tukey’s multiple comparison test were performed. Differences of p > 0.05 are not significant and not shown on the graph. p values between 0.01–0.05 are marked as significant (*), 0.001–0.1 marked as very significant (**) and p < 0.001 marked as extremely significant (***). (**b**) The dose–response plots obtained for the compounds and extracts in an HIV integrase enzyme assay. The % enzyme activity levels were derived from the absorbance values of the experimental sample compared to the untreated (control) samples. The log[compound] is plotted against the % IN enzyme activity. A non-linear regression analysis was used to calculate the IC_50_ values for the compounds of interest. Data manipulation was performed as described in the methodology.

*In vitro* viability testing has become an integral step in modern drug discovery as it characterises the toxic potential of a compound and provides predictive evidence of its safety index thus reducing attrition rates in pharmaceutical development^44,45^. Hence, the crude methanolic extract and all the compounds isolated from it (Fig. 1) were tested for cytotoxicity against the HeLa cell line using a resazurin-based cell viability assay. The inherent reducing potential of viable cells converts resazurin to resorufin (detectable by fluorimetry), and is considered as one of the most cost-effective method of determining cytotoxicity^46^. The activity of untreated HeLa cells was normalized to 100% activity and all samples were compared to the normalized control. The crude methanolic extract showed no effect on the cell viability (116.1%, Fig. 2a). Friedelin, *O*-acetyl-aleuritolic acid, 3-*O*-acetyl-erythrodiol, Stigmasterol, Friedelane-3-one-28-al and Stigmasta-4,22-diene-3-one also show no significant change in their viability, with mean cell viability values ranging from 102.3% to 118.3% (Fig. 2a). For methylgallate it showed a cell viability of 83.77%, which represents a significant reduction in the cell viability compared to all the other compounds as well as the methanol extract (Fig. 2a). This indicates that methylgallate does show some toxicity towards the HeLa cells.

### Methygallate is the primary compound responsible for the *in vitro* anti-HIV properties of *A. cardifolia*

With the increased knowledge about the catalytic mechanism of HIV IN in the recent past, considerable progress to discover potential inhibitors from natural compounds has been made. The crude methanolic extract and all the isolated compounds from the *A. cordifolia* methanolic crude extract were tested for anti-HIV IN activity. From these samples, 3-*O*-acetyl-aleuritolic acid, methylgallate, stigmasterol and the crude methanolic extract exhibited activity against HIV-1 IN (Fig. 2b). The IC_50_ of the crude extract was found to be 8.5 ng/ml. Methylgallate, stigmasterol and 3‐*O*-acetyl aleuritolic acid exhibited IC_50_ values of 3.7, 20.5 and 83.7 nM, respectively.

Interestingly, the IC_50_ of _L_-chicoric acid was found to be higher (IC_50_ = 5.9 nM) than that obtained with methylgallate. Thus, methylgallate might be the principle chemical constituent that is responsible for the anti-HIV IN activity of the methanolic *A. cordifolia* stem bark extract. In addition, the isolated compounds were also screened for potency against HIV-1 PR and found to be inactive. From the literature, crude aqueous extract from *A. cordifolia* seeds has also been shown to possess moderate *in vitro* inhibitory potency against HIV-1 RT with EC_50_ values of <0.01–0.03 mg/ml^25^.

### Methylgallate inhibits HIV-1 IN by chelating the active site Mg^2+^ cofactor

**Figure 3:**
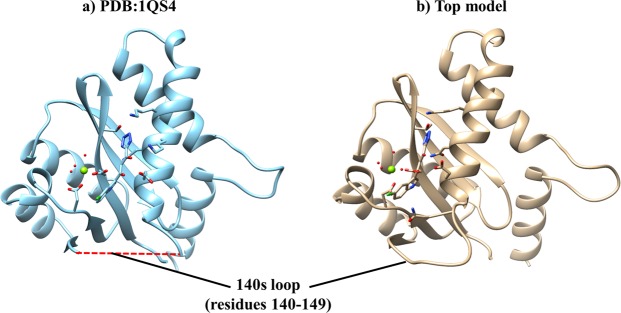
HIV-1 IN CCD homology modeling. (**a**) Template used for modeling studies with missing residues in the 140 s loop (dotted red line) bound to a co-crystallized 5-CITEP inhibitor. (**b**) Top model selected for docking studies with a complete 140 s loop. Shown in blue sphere is Mg^2+^ cofactor essential for catalytic mechanism during proviral cDNA-host genome integration. The red spheres are the metal-coordinating water molecules.

The availability of good quality and complete structural information is vital for structure-based drug design and molecular dynamics studies. Due to the missing residues in the available HIV IN structures in the PDB database, homology modelling was performed with the same target sequence as that of the recombinant protein used in the *in vitro* HIV-IN experiments. A near-native complete structure was obtained with a z-DOPE score of −1.48 as compared to that of the template (1QS4) of −1.68. Z-Dope score is an atomic distance-displacement statistical potential that evaluates how close a model is compared to the native structure^32,47^ with a preferred value of < −0.5. Table 1 shows a summary of the validation results from different quality evaluation webservers. All the results gave consistently good quality scores for the top model as to those of the template. Figure 3 shows the structural fold of both the template (1QS4) and the top model with a bound inhibitor on the active site as well as the catalytic Mg^2+^.

**Table 1:** HIV-1 IN CCD best homology model validation results from different structure quality assessment tools.

	Template: 1QS4	Top model
z-DOPE	−1.64	−1.48
Verify3D	94.77	92.63
ProSA	−7.45	−8.63
QMEAN	0.87	0.84
Ramachadran (Favoured)	99.30	99.45
GA341	1.00	1.00
Predicted native overlap	0.96	0.94

Docking results indicated that out of the six compounds from *A. cordifolia*, only methylgallate had stronger binding affinity to HIV-1 IN compared to _L_-chicoric acid (Table 2). A protein-ligand interaction analysis was performed to understand its mechanism of action. Despite methylgallate being smaller compared to _L_-chicoric acid, it exhibited a similar binding pattern involving key residues responsible for the enzyme’s catalytic mechanism. Interestingly, methylgallate was found to mimic the central core of _L_-chicoric acid involved in HIV-IN inhibition.

**Table 2:** Docking results from 100 independent runs per compound with HIV-1 IN CCD.

Compound name/ID	Docking energy (kcal/mol)	Inhibition constant (μM)	Cluster occupancy^a^
Stigmasterol	−4.32	25.97	67
Stigmasta-4,22-dien-3-one	−4.42	25.43	83
Friedelin	−3.82	30.72	89
Friedelane-3-one-28-al	−3.97	30.56	85
3-*O*-acetyl erythrodiol	−5.08	15.15	74
Methylgallate*	−5.73	12.62	100
ZINC77441**	−5.99	−11.65	92
ZINC1576053**	−6.08	−11.12	93
ZINC407934**	−6.20	−10.86	91
ZINC44199891**	−6.17	−11.03	100
_L_-chicoric acid^Δ^	−5.56	−12.87	96

^a^Number of individuals out of 100 in the top-ranked cluster. *A.cordifolia* most potent anti-HIV IN compound* and its analogues** from ZINC database. ^Δ^Positive control.

As there was a strong correlation between the *in vitro* and *in silico* anti-HIV-1 IN activity results, 127 methylgallate analogues from ZINC were obtained. Four of the analogues had binding affinities higher than that of methylgallate (Fig. S1). All the identified hits had good drug-likeness properties and passed the PAINS filter (Table 3). The PAINS filter flags chemical compounds containing substructures that cause them to promiscuously bind to numerous proteins rather than the desired drug target^48^.

**Table 3:** Drug like properties and PAINS filtering of methylgallate and its ZINC analogues.

Compound ID	Chemical formula	Lipinski’s rule of five (RO5)					PAINS
		Mol. Wt	HbA	HbD	nRB	LogP	
Methylgallate	C_8_H_8_O_5_	184.04	5	3	2	1.26	Pass
ZINC77441	C_15_H_14_O_5_	259.97	5	0	5	1.84	Pass
ZINC1576053	C_13_H_10_O_5_	235.97	5	0	3	2.18	Pass
ZINC407934	C_10_H_12_O_5_	199.97	5	0	3	2.17	Pass
ZINC44199891	C_17_H_18_O_4_	267.98	4	0	7	1.59	Pass
_L_-chicoric	C_22_H_18_O_12_	474.08	12	6	11	1.18	Pass

**Figure 4:**
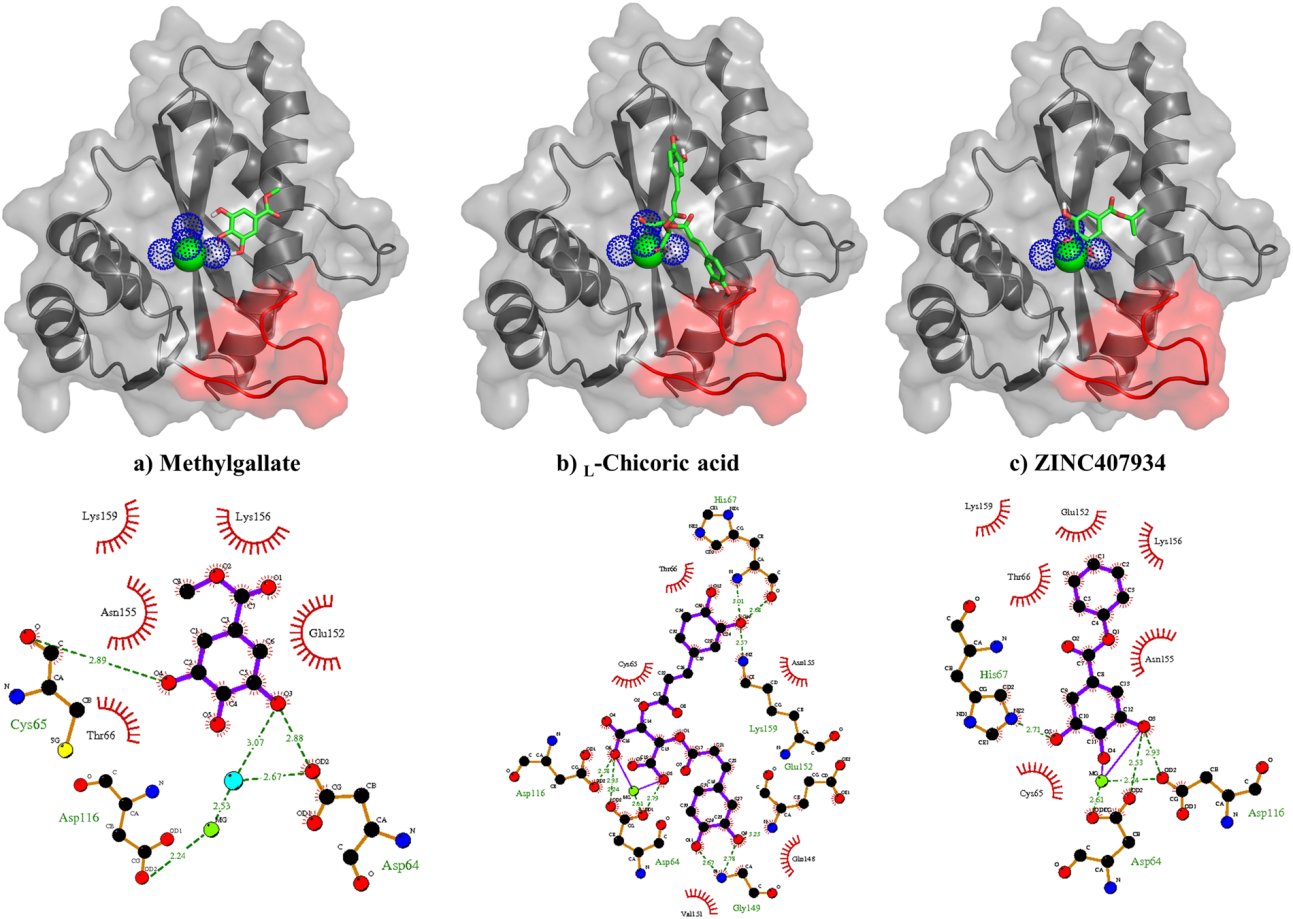
Protein-ligand binding pose and interaction. HIV-IN complete CCD bound to (**a**) methylgallate from *A. cordifolia* and (**b**) _L_-chicoric acid and (**c**) ZINC407934. Corresponding images show the important residues for coordinating the catalytic Mg^2+^ as well as interacting with the different ligands. Shown in blue dotted green lines are hydrogen bonds while the radiating residues show the residues forming hydrophobic interactions. The red loop shows the 140 s loop.

Existing IN drugs including _L_-chicoric acid, possess a characteristic *β*-diketo moiety which chelates the metal co-factor thus selectively inhibiting the strand transfer reaction^29^. From the docking results, methylgallate, its four ZINC analogues and _L_-chicoric acid formed hydrogen bonds with the Mg^2+^ cofactor either directly or indirectly (via coordinating water molecules), making it unavailable for catalysis (Fig. 4). The presence of hydroxyl groups in methylgallate and its ZINC analogues that are close enough to interact with the metal cofactor was essential for the hydrogen bond formation. This might explain the observed inactivity with the rest of the compounds from *A. cordifolia* which had mainly hydrophobic cores (Fig. S2). A similar observation regarding the importance of hydroxyl groups on IN inhibitory activity of chalcones and homoisoflavonoids from *Caesalpinia sappan* has been reported^49^. Besides the hydrogen bonds by Asp64 and Asp116, methylgallate and the ZINC hits formed additional hydrogen bonds with active site residues (Fig. 4). In methylgallate and ZINC1576053, one of the vicinal hydroxyl groups in the compounds formed a hydrogen bond with Cys65 and His67 respectively. ZINC407934 formed two hydrogen bonds; one with a hydroxyl group and the other with its alkoxy oxygen of the ester group with Asn155. These additional hydrogen bonds might explain its strong interaction of −6.2 kcal/mol compared to methylgallate.

Additional hydrophobic contacts with similar residues common across the different ligands were observed. These residues include Cys65, Glu152, Asn155, Lys156, and Lys159. Of particular interest is the ability of these ligands to interact with the thiol group of Cys65 which is known to be an efficient nucleophile as it can be oxidized with ease^50^. Unique to _L_-chicoric acid was the ability to interact with Gln148 found in the 140 s loop facing the active site. This interaction was not observed with the other compounds mainly because of their small size. Molecular modeling studies with 5-CITEP (1QS4 co-crystalized ligand) show similar binding modes as the current ligands and interacted with the identified residues^51^. A structure-activity relationship study of HIV-1 IN inhibitors indicated that for optimum interaction of _L_-chicoric acid, it has to adopt a pose in which the two aromatic rings form a 90° angle^50^.

**Figure 5:**
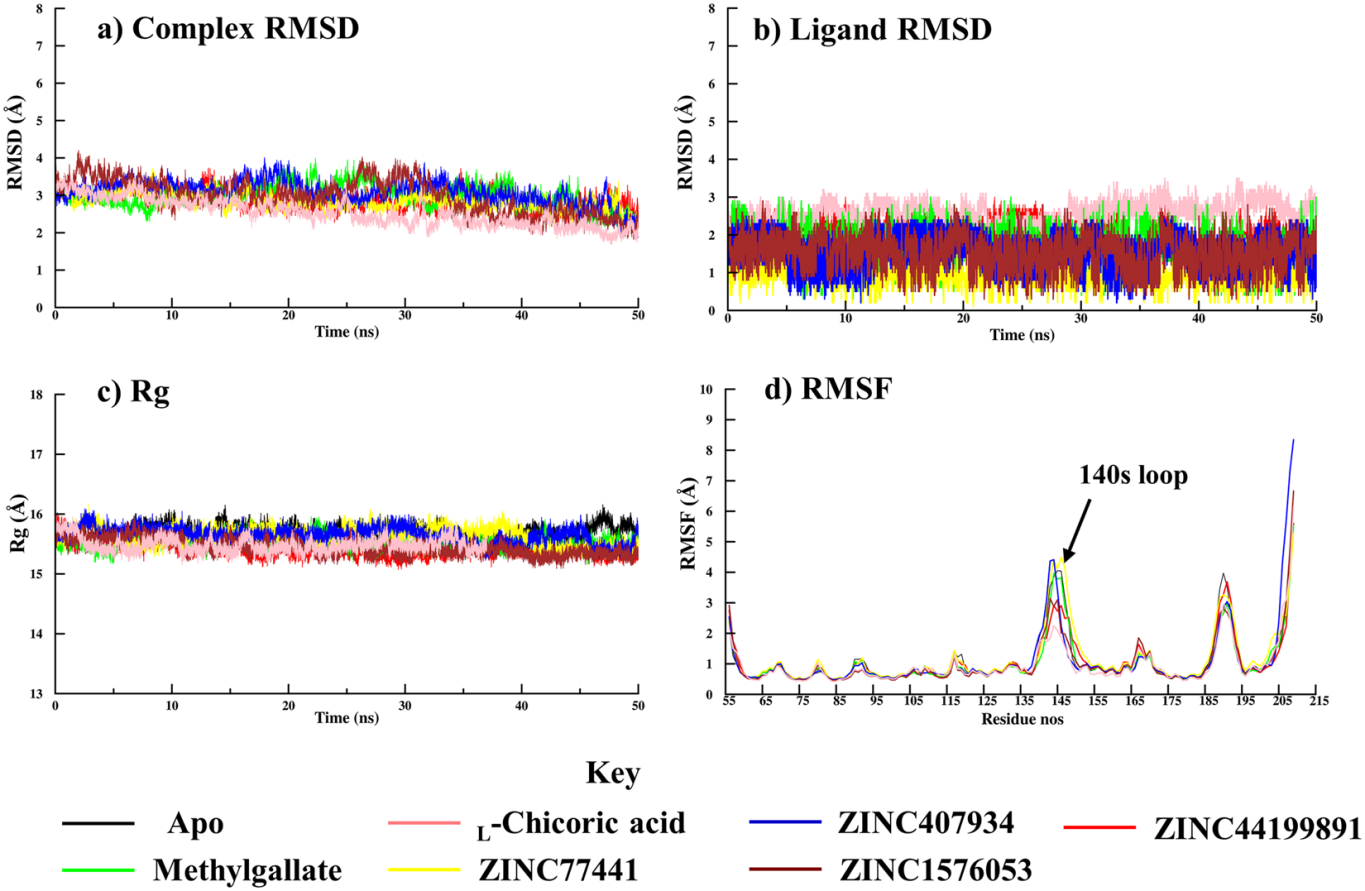
Dynamic properties of both the apo form and ligand bound HIV-IN CCD over a simulation period of 100 ns. (**a**,**b**) The global stability of the different systems as determined by complex and ligand Root Mean Square Deviation (RMSD). (**c**) The compactness of the systems by Radius of Gyration. (**d**) Local fluctuations of the individual residues by Root Mean Square Fluctuations (RMSF).

Additional studies involving MD simulations were performed to assess the behaviour of the protein ligand complexes relative to the apoprotein. For each protein system, 50 ns simulations were prepared. The dynamic stability of each protein-ligand complexe was assessed using backbone Root Mean Square Deviation (RMSD), Root Mean Square Fluctuation (RMSF) and radius of gyration (Rg). Both the apo and ligand-bound forms of the enzyme displayed similar conformational stability during the molecular dynamic simulations (Fig. 5). C^α^ RMSD analysis showed that the systems converged around 3–5 Å (Angstrom) immediately after equilibration (Fig. 5a). Additionally, the ligands maintained same poses throughout the MD simulations (Fig. 5b). A similar observation regarding global stability was made with Rg where protein-ligand complexes were found to maintain a radius between 15 and 16 Å during simulations (Fig. 5c). RMSF calculations were performed to determine the fluctuations within the residues. All the catalytic triad residues and their neighbouring residues were stable (Fig. 5d). However, prominent fluctuations were observed in the 140 s loop residues in the apo form and when the protein was bound to methylgallate (Fig. 5d). A significant reduction in the fluctuation of this loop was observed when the protein was bound to _L_-chicoric acid, mainly because of the observed interactions between the ligand and Gln148 (Fig. 4b). As expected, high fluctuations similar to those in the apo form were observed in the methylgallate, ZINC77441, ZINC407934 bound proteins as no interaction between these ligands and the 140 s loop were observed.

## Conclusion

According to the current antiretroviral guidelines, integrase strand transfer inhibitors (InSTIs) are considered the cornerstone of the HAART regimens against HIV/AIDS because they are highly effective and well tolerated compared to RT and PR inhibitors^52,53^. Considering the limited number of FDA approved InSTIs and the rise of viral mutants exhibiting resistance to existing drugs, the discovery of novel InSTIs becomes imperative. So far, CCD viral mutants with G148H, N155H and G148H/G140S polymorphisms have been reported and have shown resistance against Elvitegravir and Dolutegravir underscoring the need for the development of novel drugs^54,55^.

Methylgallate was found to be an important inhibitor of HIV-IN with its IC_50_ value 3.7 nM, which is lower than that of the positive control i.e. _L_-chicoric acid (IC_50_ = 5.9 nM). This not only confirms that methylgallate could be used as lead for new HIV drugs, but also that *A. cordifolia* is a potential source for HIV drugs discovery. Both the *in vitro* and *in silico* results presented here show that methylgallate is a possible platform for the development of novel HIV-1 IN drugs. Additional downstream optimization studies involving structure-activity relationships are needed to improve the potency of the identified compounds. One of the possibilities to enhance their potency is the utilization of synthetic chemical approaches to increase their size in order to interact with the catalytic 140 s loop. Considering the vital role played by hydroxyl or ester groups that interact with the metal cofactor, the incorporation of such chemical moities would also be valuable.

## Supplementary information

Supplementary information

## Data Availability

The authors confirm that the data supporting the findings of this study are available within the article and its Supplementary Information.
