# Supplementary material for: Anti-HIV-1 integrase potency of methylgallate from Alchornea cordifolia using in vitro and in silico approaches
Source: Sci Rep. 2019 Mar 18;9:4718. doi: 10.1038/s41598-019-41403-x (PMC6423119; doi:10.1038/s41598-019-41403-x)

Anti-HIV-1 integrase potency of methylgallate from *Alchornea cordifolia* using *in vitro* and *in silico* approaches

Xavier Siwe Noundou[1,4*], Thommas M. Musyoka[2], Vuyani Moses[2], Derek T. Ndinteh[3], Dumisani Mnkandhla[4], Heinrich Hoppe[4], Özlem Tastan Bishop[2*] & Rui W.M. Krause[1*]

[1]Department of Chemistry, Rhodes University, Grahamstown 6140, South Africa

[2]Research Unit in Bioinformatics (RUBi), Department of Biochemistry and Microbiology, Rhodes University, Grahamstown 6140, South Africa

[3]Department of Applied Chemistry, University of Johannesburg, Doornfontein, Johannesburg 2028, South Africa

[4]Department of Biochemistry and Microbiology, Rhodes University, Grahamstown 6140, South Africa

Correspondence and requests for materials should be addressed to X.S.N, Ö.T.B and RWMK. (email: xavsiw@gmail.com, O.TastanBishop@ru.ac.za and r.krause@ru.ac.za)

Supplementary Information

Figure S1. The structures of methylgallate analogs from the ZINC database.

Figure S2. HIV-1 CCD domain interaction with compounds from *A. cordifolia* that showed no *in vitro* inhibitory potency.

Figure S1

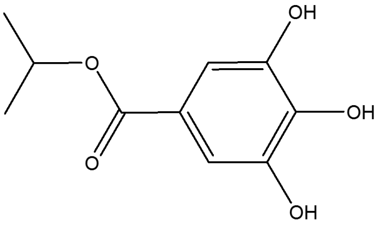

ZINC407934

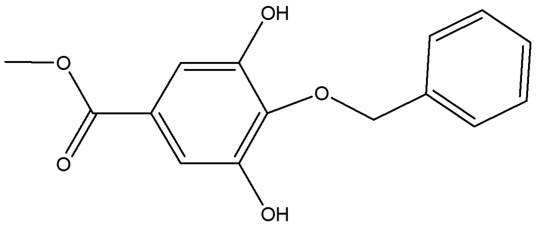

ZINC77441

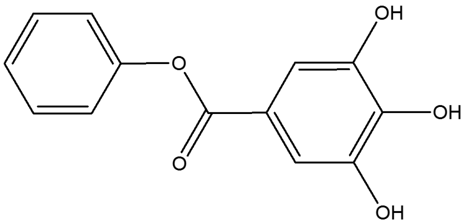

ZINC1576053

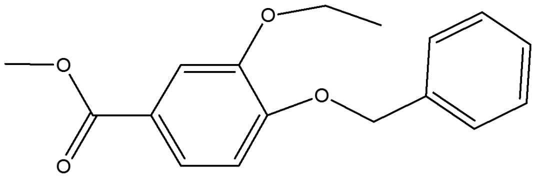

ZINC44199891

Figure S2

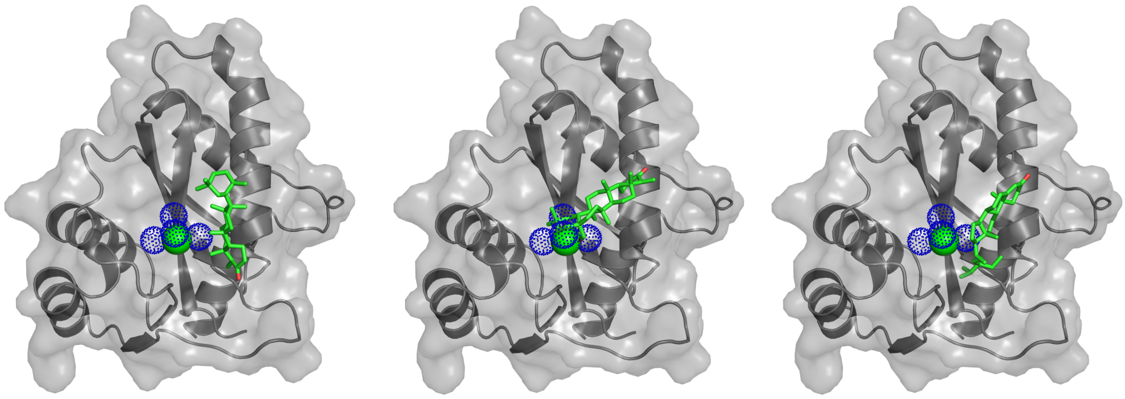

a) Friedelin **b) Friedelane-3-one-28-al** **c) Stigmasta-4,22-dien-3-one**

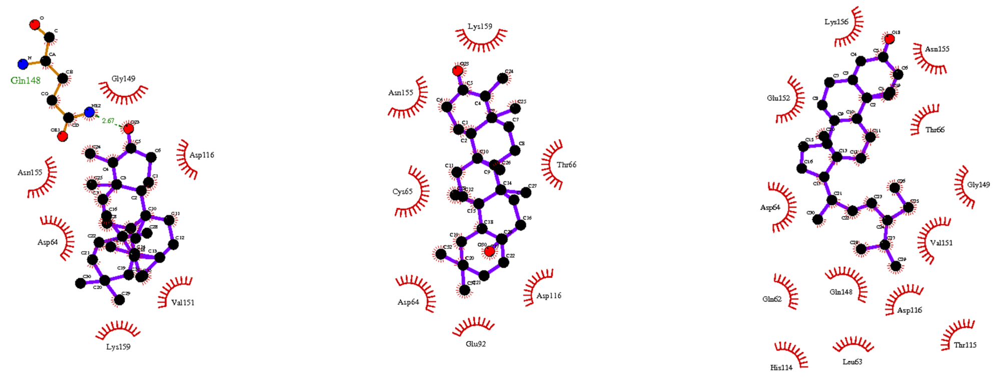

Supplement: Supplementary file 1 — Supplementary information [file 41598_2019_41403_MOESM1_ESM.pdf]
